# Supplementary material for: Ventromedial prefrontal neurons represent self-states shaped by vicarious fear in male mice
Source: Nat Commun. 2023 Jul 3;14:3458. doi: 10.1038/s41467-023-39081-5 (PMC10318047; doi:10.1038/s41467-023-39081-5)
Supplement: Supplementary file 1 — Supplementary Information [file 41467_2023_39081_MOESM1_ESM.pdf]

Supplementary Information for

Ventromedial prefrontal neurons represent self-states shaped by vicarious fear in male mice

Ziyan Huang, Myung Chung, Kentaro Tao, Akiyuki Watarai, Mu-Yun Wang, Hiroh Ito, Teruhiro Okuyama

This PDF file includes:

Supplementary Figure 1 to S11
Supplementary Table

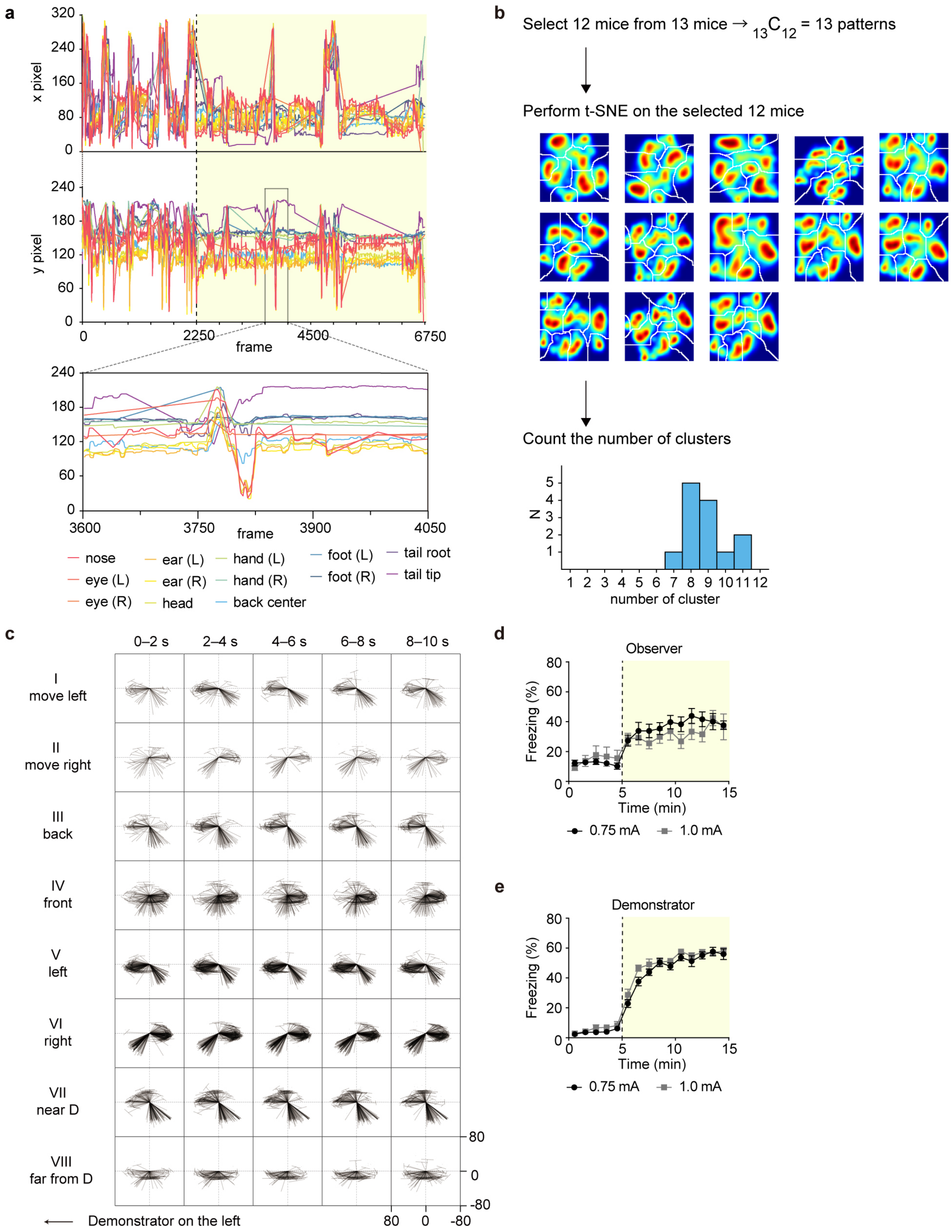

a
x pixel
y pixel
frame
nose
eye (L)
eye (R)
ear (L)
ear (R)
head
hand (L)
hand (R)
back center
foot (L)
foot (R)
tail root
tail tip
b
Select 12 mice from 13 mice → $_{13}C_{12}$ = 13 patterns
Perform t-SNE on the selected 12 mice
Count the number of clusters
N
number of cluster
c
0–2 s
2–4 s
4–6 s
6–8 s
8–10 s
I move left
II move right
III back
IV front
V left
VI right
VII near D
VIII far from D
Demonstrator on the left
d
Observer
Freezing (%)
Time (min)
0.75 mA
1.0 mA
e
Demonstrator
Freezing (%)
Time (min)
0.75 mA
1.0 mA

Supplementary Fig. 1. Details of DeepLabCut tracking and behavioral clustering.

a, Results of t-SNE embedding of 13 body-point tracking experiments using DeepLabCut (data from n = 13 mice, 1,170 bouts total). Representation of 13 body-point tracking from the x and y axis with linear interpolation. **b**, Behavioral cluster classification by t-SNE using 12 out of 13 mice data. We confirmed that 8 was the most adequate number of clusters, as 8 clusters were most commonly found in 13 patterns of 12 randomly selected datasets and the clusters were not over-subdivided. **c**, Diagram of the skeleton using four points (left ear, right ear, back center, and tail root) with the back center point aligned to (0,0) for every 2 seconds. **d**, Freezing rate of observer mice with shock intensity of 0.75 mA and 1.0 mA (n = 10 mice each, two-way repeated ANOVA, $F(14, 252) = 1.30$, $P = 0.21$). **e**, Freezing rate of demonstrator mice in with shock intensity of 0.75 mA and 1.0 mA (n = 10 mice each, two-way repeated ANOVA, $F(14, 252) = 0.86$, $P = 0.60$). Data are presented as mean ± SEM.

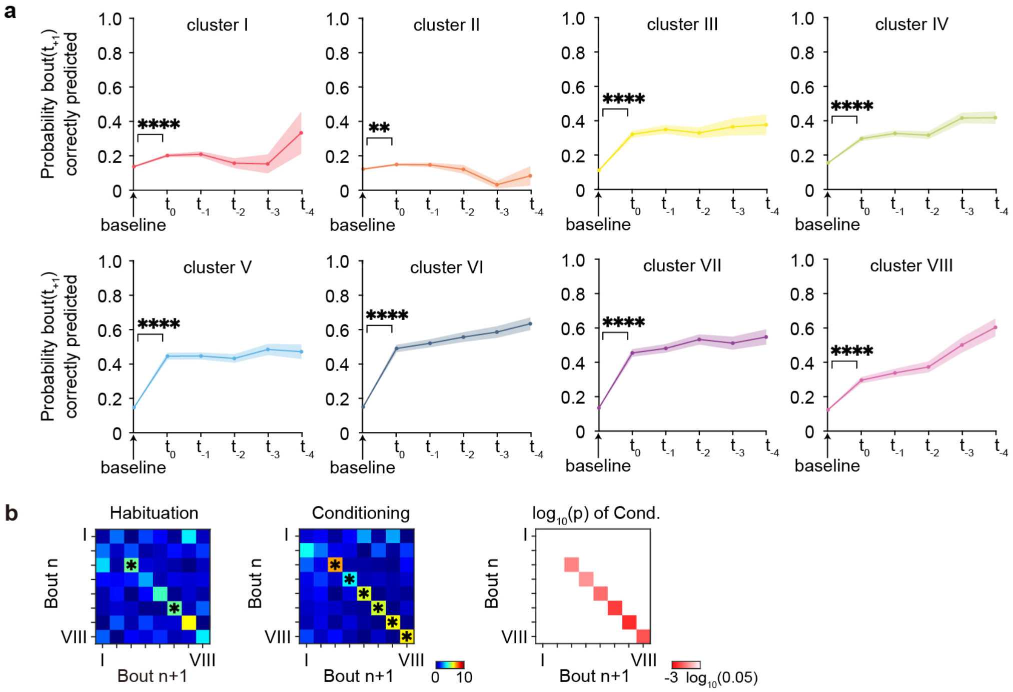

Supplementary Fig. 2. Cluster transition.

a, The accuracy of the prediction of the next bout state t_{+1} with a zeroth-order model (based on the distribution of the clusters, baseline), a first-order model (the current bout state is known, t_0), a second-order model (the current bout state and the previous bout state are known, t_{-1}), etc. The accuracies were compared between two successive models for each cluster (e.g., baseline vs. t_0, t_0 vs. t_{-1}; $P < 0.025/5 = 0.005$, paired two-tailed t-test, Bonferroni correction). Cluster I: baseline vs. t_0, **** $P < 0.0001$, Cluster II: baseline vs. t_0, ** $P = 0.0015$, Cluster III: baseline vs. t_0, **** $P < 0.0001$, Cluster IV: baseline vs. t_0, **** $P < 0.0001$, Cluster V: baseline vs. t_0, **** $P < 0.0001$, Cluster VI: baseline vs. t_0, **** $P < 0.0001$, Cluster VII: baseline vs. t_0, **** $P < 0.0001$, Cluster VIII: baseline vs. t_0, **** $P < 0.0001$. Data are presented as mean ± SEM. **b**, Left and center, transition probability divided by the chance level (the mean transition probability obtained from the permutation test) from bout n to bout n+1 during the habituation (left) and conditioning periods (center). Significant transitions calculated by the permutation test are marked (one-sided, $P < 0.05$). Right, the common logarithm of the p-value of each significant transition during the conditioning period.

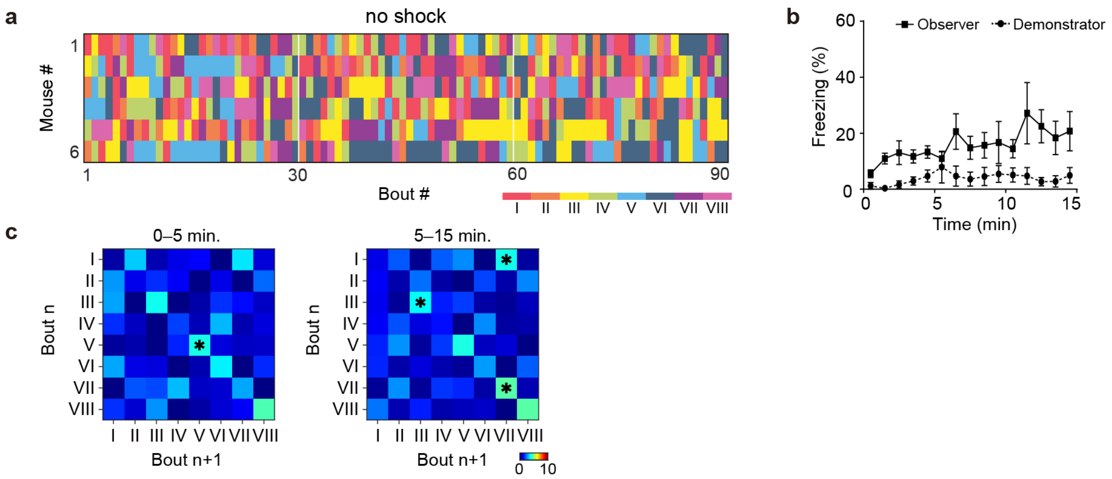

Supplementary Fig. 3. Behavioral pattern analysis of the observer mice while no shock was applied to the demonstrator mice.

a, Behavioral sequence of each mouse during the task (n = 6 mice). **b**, Freezing rate of the observer (n = 6 mice, one-way repeated-measures ANOVA, F = 1.77, P = 0.21) and the demonstrator (n = 6 mice). Data are presented as mean ± SEM. **c**, Transition probability divided by the chance level (the mean transition probability obtained in the permutation test) from bout n to bout n+1 in the first 5 minutes (left) and the last 10 minutes (right). Significant transitions calculated by the permutation test are marked (one-sided, * P < 0.05).

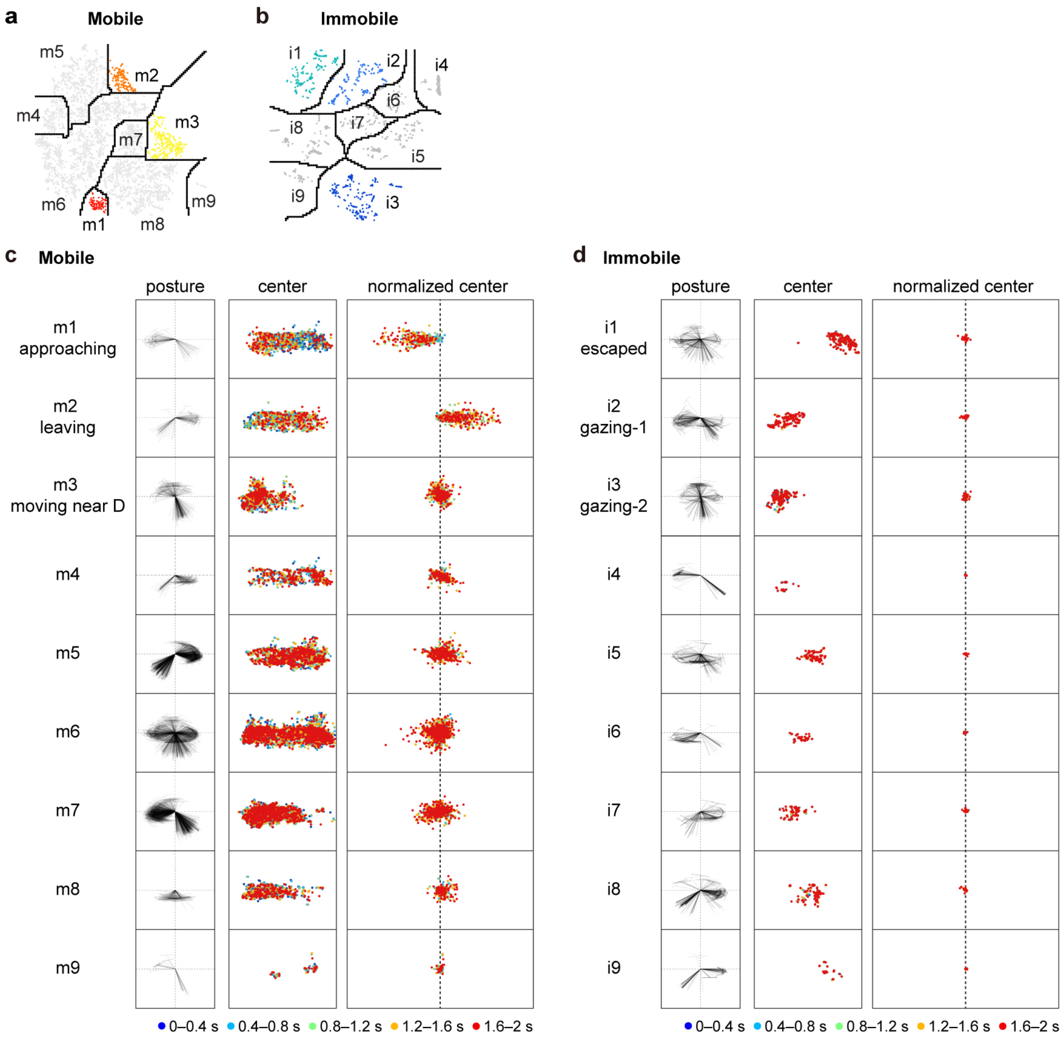

Supplementary Fig. 4. Details of component classification.

a, Results of t-SNE embedding of 13 body-point tracking experiments using DeepLabCut (data from n = 13 mice, mobile bouts: 4,335 bouts). **b,** Results of t-SNE embedding of 13 body-point tracking experiments using DeepLabCut (data from n = 13 mice, immobile bouts: 1,515 bouts). **c**,**d**, c: Mobile bouts. d: Immobile bouts. Left, diagram of the skeleton in the 2-s bouts using four points (left ear, right ear, back center, tail root) with the back center point aligned to (0,0) (xpixel: -80 to 80, ypixel: -80 to 80). Center and right, the position of the back center in the chamber (center, xpixel: 0 to 320, ypixel: 0 to 240) and back center points with the position of the first frame of each bout (75 frames) were set to (0,0) (right, xpixel: -280 to 280, ypixel: 0 to 240).

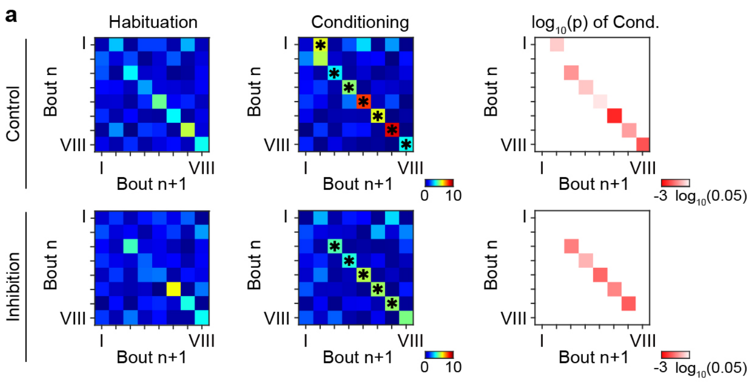

Supplementary Fig. 5. Cluster transition in optogenetic inhibition of the vmPFC.

a, Left and center, transition probability divided by the chance level (the mean transition probability obtained in the permutation test) from bout n to bout n+1 during the habituation (left) and conditioning periods (center). Significant transitions calculated by the permutation test were marked (one-sided, $P < 0.05$). Right, the common logarithm of the p-value of each significant transition during the conditioning period.

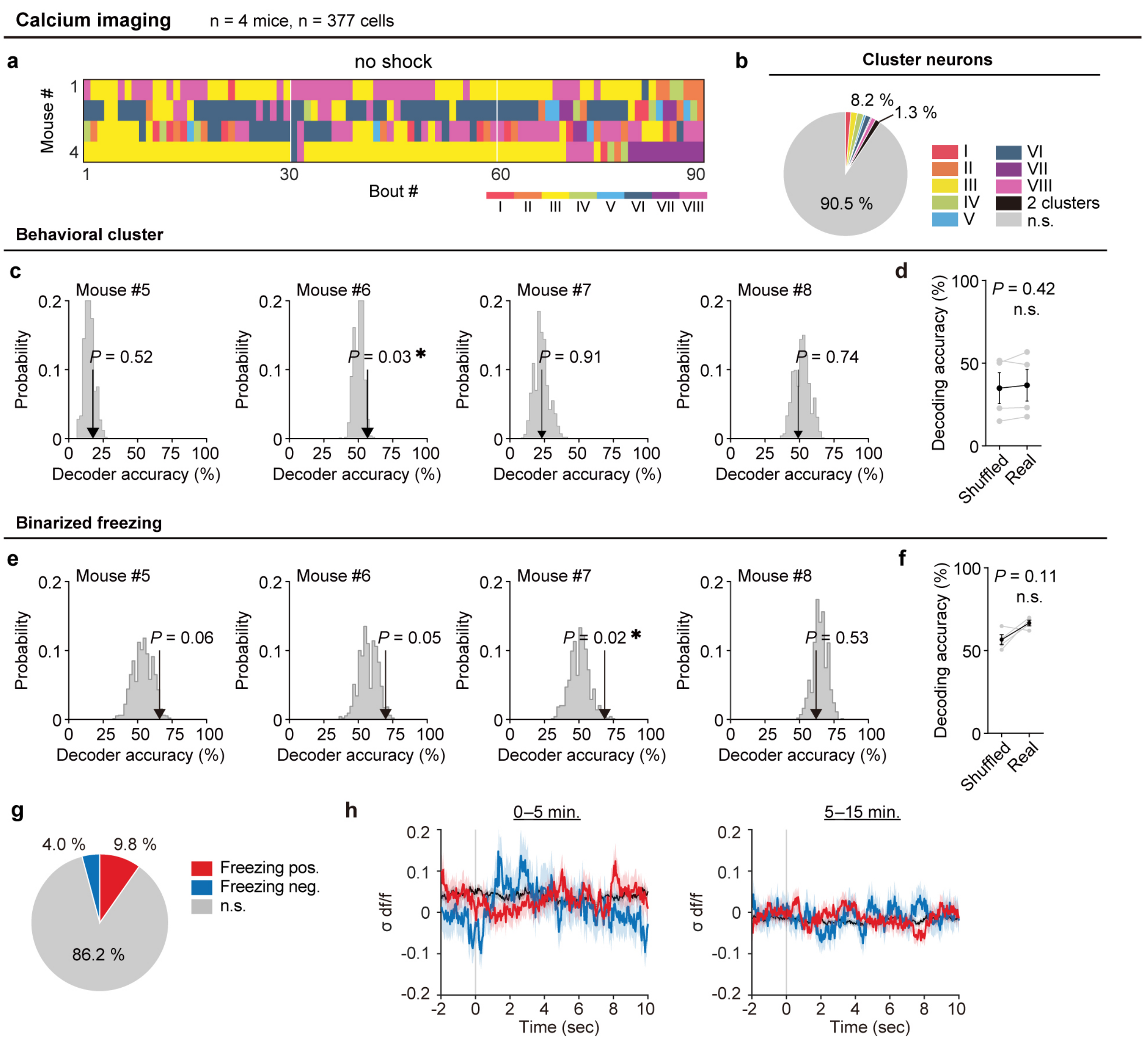

Supplementary Fig. 6. Ca^{2+} imaging of the observer mice while no shock was applied to the demonstrator mice.

a, Behavioral sequence of each mouse during the task (n = 4 mice). **b**, Proportion of cluster-specific neurons (n = 4 mice, n = 377 cells). Cells responded to 2 clusters include 1 neuron to I and II, 1 neuron to I and IV, 2 neurons to IV and V, and 1 neuron II and V. **c**, Histogram of the decoding accuracy of the behavioral cluster using shuffled data for each mouse. Arrows indicate the accuracy and one-sided p-value of the real data. **d**, Decoding accuracy of the behavioral cluster compared to the shuffled data (n = 4 mice, P = 0.097, two-sided paired t-test). **e**, Histogram of the decoding accuracy of the binarized freezing states using shuffled data of each mouse.

Arrows indicate the accuracy and one-sided p-value of the real data. **f**, Decoding accuracy of the binarized freezing states compared to the shuffled data (n = 4 mice, P = 0.11, two-sided paired t-test). **g**, Proportion of neurons significantly correlated with self-freezing rate. **h**, Bout-averaged responses of self-freezing correlated neurons. * P < 0.05, n.s. not significant. Data are presented as mean ± SEM (error bars and shadows).

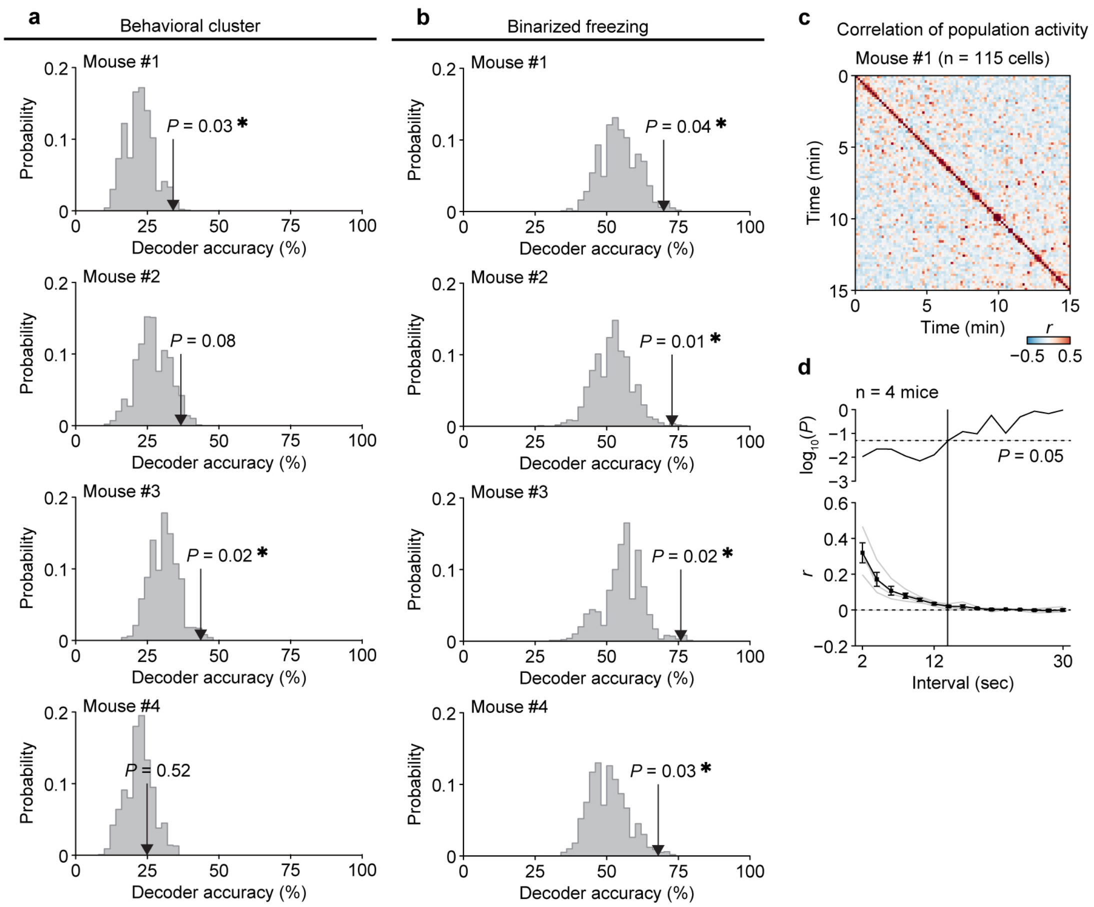

Supplementary Fig. 7. Decoder accuracy and the correlation of population activity in the vmPFC.

a, Histogram of the decoding accuracy of the behavioral cluster using shuffled data for each mouse. Arrows indicate the accuracy and one-sided p-value of the real data. **b**, Histogram of the decoding accuracy of the binarized freezing states using shuffled data of each mouse. Arrows indicate the accuracy and one-sided p-value of the real data. **c**, Correlation of population activity of mouse #1. **d**, Correlation of population activity during the conditioning period by interval and the common logarithm of the two-sided p-value (n = 4 mice). * $P < 0.05$. Data are presented as mean ± SEM.

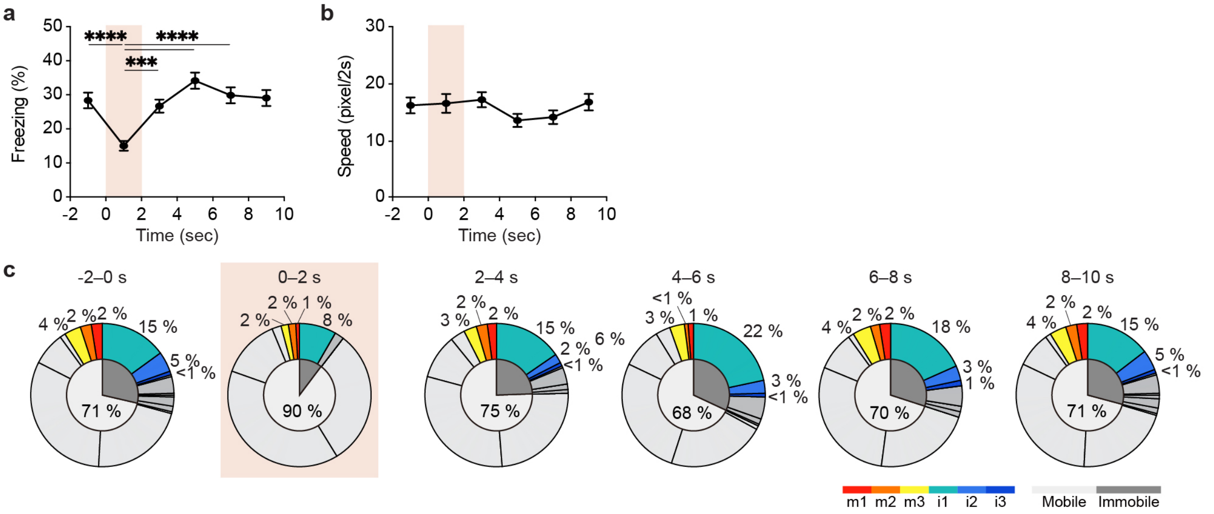

Supplementary Fig. 8. Behavior description of the vmPFC calcium recording mice.

a, Average freezing rate during the conditioning period (n = 4 mice, one-way ANOVA for time point -2–0 s, 0–2 s, 2–4 s, 4–6 s, 6–8 s, $F(4,1191) = 16.52$, **** $P < 0.0001$, Post-hoc test, Turkey-Kramer test; -2–0 s vs. 0–2 s, $P < 0.0001$, 0–2 s vs. 2–4 s, $P = 0.0008$, 0–2 s vs. 4–6 s, $P < 0.0001$, 0–2 s vs. 6–8 s, $P < 0.0001$). **b,** Average speed of the body center along x coordinate during the conditioning period (n = 4 mice, one-way ANOVA for time point -2–0 s, 0–2 s, 2–4 s, 4–6 s, 6–8 s, $F(4,1191) = 1.438$, $P = 0.22$). **c**, Proportion of components in the -2–0 s, 0–2 s, 2–4 s, 4–6 s, 6–8 s, and 8–10 s during the conditioning period (n = 4 mice). *** $P < 0.001$, **** $P < 0.0001$. Data are presented as mean ± SEM.

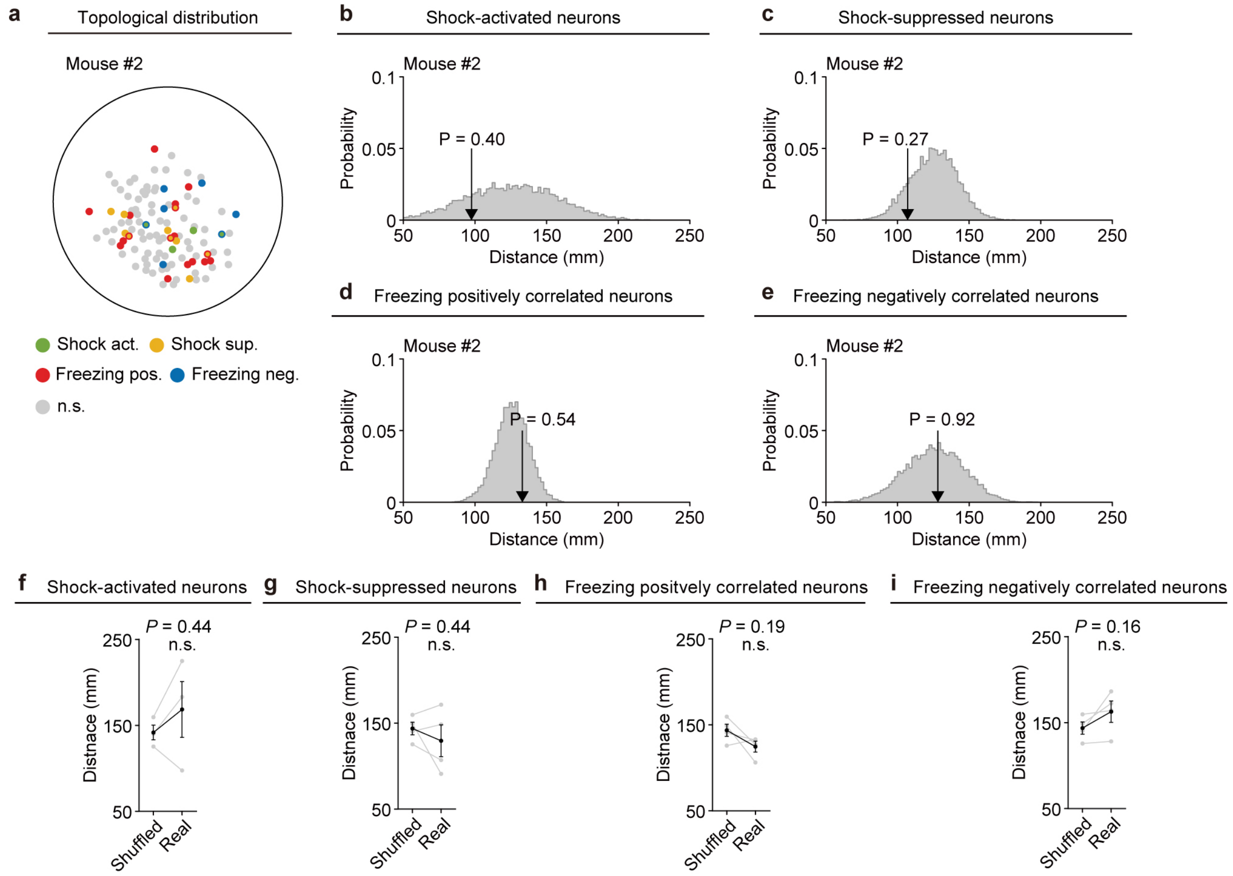

j

Freezing \ Shock	Act.	n.s.	Sup.
Pos.	0 % (n = 0)	9.3 % (n = 33)	3.1 % (n = 11)
n.s.	2.0 % (n = 7)	62.8 % (n = 223)	4.5 % (n = 16)
Neg.	2.3 % (n = 8)	15.8% (n = 56)	0.3 % (n = 1)

Supplementary Fig. 9. The topological distance among annotated neurons.

a, Topological distribution of self-freezing and other-shock significant neurons of mouse #2. **b**, Histogram of the distances between shock-activated neurons using shuffled data of mouse #2. Arrow indicates the accuracy and one-sided p-value of the real data. **c**, Histogram of the distance between shock-suppressed neurons using shuffled data of mouse #2. Arrow indicates the accuracy and one-sided p-value of the real data. **d**, Histogram of the distances between neurons positively correlated with freezing using shuffled data of mouse #2. Arrow indicates the accuracy

and one-sided p-value of the real data. **e**, Histogram of the distance between neurons negatively correlated with freezing using shuffled data of mouse #2. Arrow indicates the accuracy and one-sided p-value of the real data. **f**, Decoding accuracy of the distances between shock-activated neurons compared to the shuffled data (n = 3 mice, P = 0.44, two-sided paired t-test). **g**, Decoding accuracy of the distances between shock-suppressed neurons compared to the shuffled data (n = 4 mice, P = 0.44, two-sided paired t-test). **h**, Decoding accuracy of the distances between neurons positively correlated with freezing compared to the shuffled data (n = 4 mice, P = 0.19, two-sided paired t-test). **i**, Decoding accuracy of distances between neurons negatively correlated with freezing compared to the shuffled data (n = 4 mice, P = 0.16, two-sided paired t-test). n.s. not significant. Data are presented as mean ± SEM. **j**, Overlap of shock-responding neurons and freezing-correlated neurons.

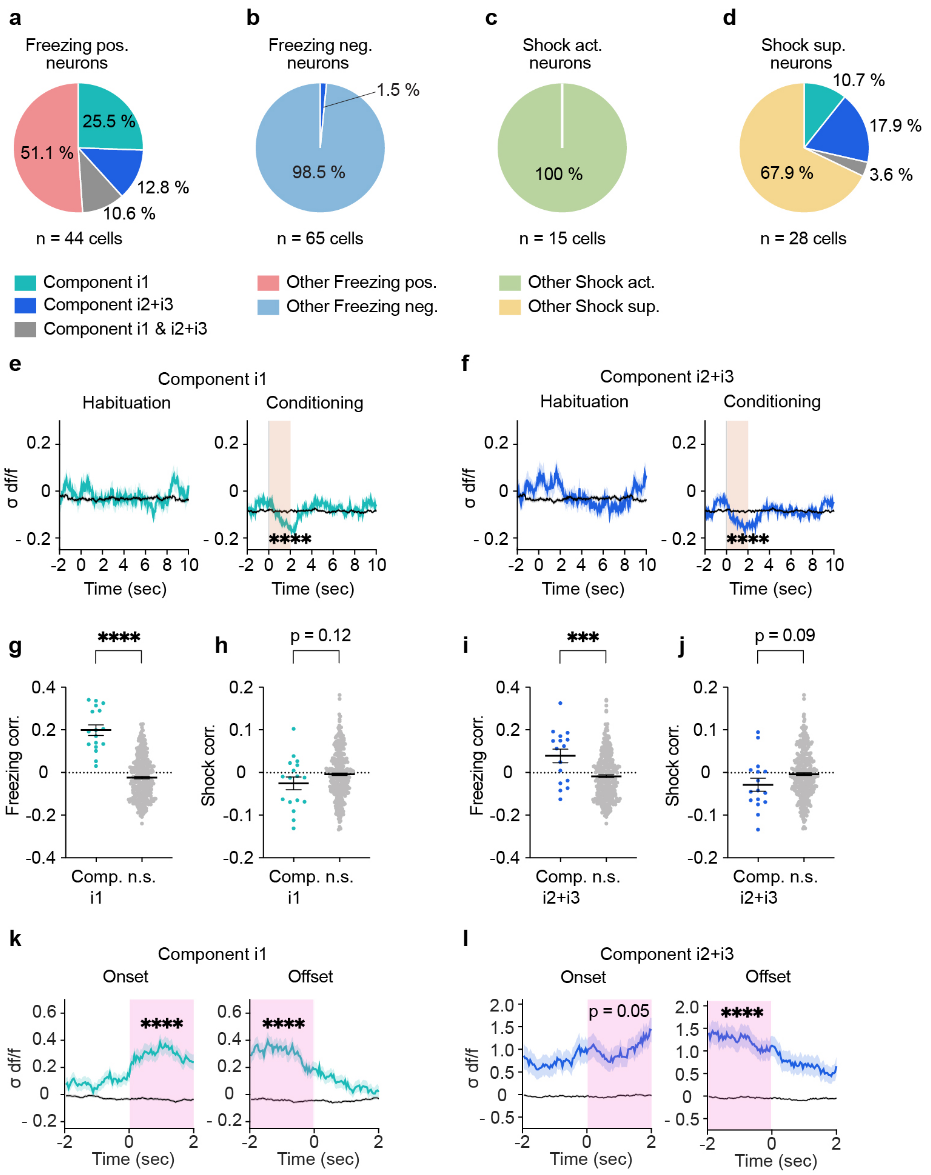

Supplementary Fig. 10. Neural properties of i1 & i2+i3 component neurons.

a, Proportion of component neurons in the self-freezing positively-correlated neurons. **b**, Proportion of component neurons in the self-freezing negatively-correlated neurons. **c**, Proportion of component neurons in the other-shock activated neurons. **d**, Proportion of component neurons in the other-shock suppressed neurons. **e,** Change in activity of i1-specific neurons during 2-s shock bouts compared to preceding 2-s interval (Wilcoxon signed-rank test, two-sided). Colored shadow in the 0–2 s time bin of conditioning period indicates shock timing. **f**, Change in activity of i2+i3-specific neurons during 2-s shock bouts compared to preceding 2-s interval (Wilcoxon

signed-rank test, two-sided). Colored shadow in the 0–2 s time bin of conditioning period indicates shock timing. **g**, Freezing correlations of i1-specific neurons (Unpaired two-sided t-test, n = 355 cells). **h**, Shock correlations of i1-specific neurons (Unpaired two-sided t-test, n = 351 cells). **i**, Freezing correlations of i2+i3-specific neurons (Unpaired two-sided t-test, n = 355 cells, P = 0.0007). **j**, Shock correlations of i2+i3-specific neurons (Unpaired two-sided t-test, n = 351 cells). **k**, Bout-averaged responses of i1 neurons aligned by the onset (Left) and at the offset (Right) of i1-specific neurons. Only the first and the last 2-s bout of repetitively appeared components were included, respectively (Wilcoxon signed-rank test, two-sided, 559 bouts). Colored shadows indicate the component i1 2-s bout. **l,** Bout-averaged responses of i2+i3 neurons aligned by the onset (Left) and at the offset (Right) of i2+i3 -specific neurons. Only the first and the last 2-s bout of repetitively appeared components were included, respectively (Wilcoxon signed-rank test, two-sided, 74 bouts). Colored shadows indicate the component i2+i3 2-s bout. **** P < 0.0001. Data are presented as mean ± SEM (error bars and shadows).

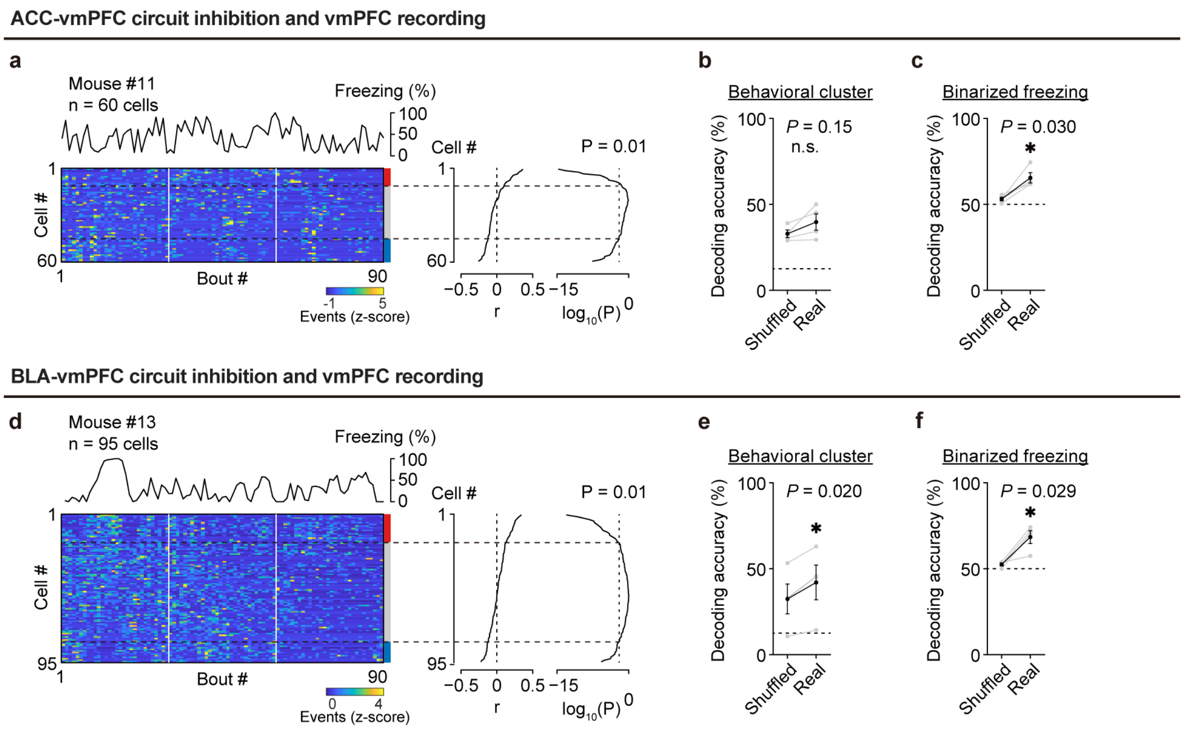

Supplementary Fig. 11. Freezing correlation and decoder accuracy of the vmPFC neurons during ACC-vmPFC and BLA-vmPFC inhibition.

a, Neural activity sorted by the degree of correlation with the self-freezing rate of mouse #11 (Pearson's correlation coefficient, two-sided). **b**, Decoding accuracy of the behavioral cluster compared to the shuffled data (n = 4 mice, P = 0.15, two-sided paired t-test). **c**, Decoding accuracy of the binarized freezing states compared to the shuffled data (n = 4 mice, * P = 0.030, two-sided paired t-test). **d**, Neural activity sorted by the degree of correlation with the self-freezing rate of mouse #13 (Pearson's correlation coefficient, two-sided). **e**, Decoding accuracy of the behavioral cluster compared to the shuffled data (n = 4 mice, * P = 0.020, two-sided paired t-test). **f**, Decoding accuracy of the binarized freezing states compared to the shuffled data (n = 4 mice, * P = 0.029, two-sided paired t-test). * $P < 0.05$, n.s. not significant. Data are presented as mean ± SEM.

Supplementary Table			
**Statistical Information for Main Figures**			
**Figure**	**Sample Size**	**Analysis**	**Statistical Test Values**<br>**(* P < 0.05, ** P < 0.01, *** P < 0.001, **** P < 0.0001)**
Figure 1b	n = 26 mice<br>· Demonstrator: 13 mice<br>· Observer: 13 mice	One-way repeated measures ANOVA<br><br>Post-hoc test: Tukey-Kramer test	(Demonstrator)<br>F(3.222, 38.66) = 71.03, **** P < 0.0001<br>5 min vs. 6 min: P = 0.058<br>5 min vs. 7 min: *** P = 0.0002<br>5 min vs. 8 min: *** P = 0.0002<br>5 min vs. 9 min: **** P < 0.0001<br>5 min vs. 10 min: **** P < 0.0001<br>5 min vs. 11 min: **** P < 0.0001<br>5 min vs. 12 min: **** P < 0.0001<br>5 min vs. 13 min: **** P < 0.0001<br>5 min vs. 14 min: **** P < 0.0001<br>5 min vs. 15 min: **** P < 0.0001<br><br>(Observer)<br>F(5.221, 62.66) = 18.50, **** P < 0.0001<br>5 min vs. 6 min: *** P = 0.0008<br>5 min vs. 7 min: ** P = 0.0012<br>5 min vs. 8 min: ** P = 0.0041<br>5 min vs. 9 min: * P = 0.013<br>5 min vs. 10 min: *** P = 0.0005<br>5 min vs. 11 min: ** P = 0.0010<br>5 min vs. 12 min: *** P = 0.0004<br>5 min vs. 13 min: *** P = 0.0001<br>5 min vs. 14 min: ** P = 0.0016<br>5 min vs. 15 min: ** P = 0.0076

Figure 1h	n = 1170 bouts<br>· cluster I: 90 bouts<br>· cluster II: 77 bouts<br>· cluster III: 121 bouts<br>· cluster IV: 225 bouts<br>· cluster V: 179 bouts<br>· cluster VI: 192 bouts<br>· cluster VII: 148 bouts<br>· cluster VIII: 138 bouts	One-way ANOVA<br><br>Post-hoc test: Tukey-Kramer test	F(7,1162) = 20.66, **** P < 0.0001<br>I vs. II: P = 0.97<br>I vs. III: **** P < 0.0001<br>I vs. IV: **** P < 0.0001<br>I vs. V: **** P < 0.0001<br>I vs. VI: **** P < 0.0001<br>I vs. VII: **** P < 0.0001<br>I vs. VIII: **** P < 0.0001<br>II vs. III: **** P < 0.0001<br>II vs. IV: **** P < 0.0001<br>II vs. V: **** P < 0.0001<br>II vs. VI: **** P < 0.0001<br>II vs. VII: **** P < 0.0001<br>II vs. VIII: **** P < 0.0001<br>III vs. IV: P > 0.99<br>III vs. V: P = 0.59<br>III vs. VI: P = 0.13<br>III vs. VII: P = 0.54<br>III vs. VIII: P = 0.78<br>IV vs. V: P = 0.21<br>IV vs. VI: * P = 0.012<br>IV vs. VII: P = 0.20<br>IV vs. VIII: P = 0.44<br>V vs. VI: P = 0.99<br>V vs. VII: P > 0.99<br>V vs. VIII: P > 0.99<br>VI vs. VII: P = 0.99<br>VI vs. VIII: P = 0.96<br>VII vs. VIII: P > 0.99
Figure 2d	n = 5850 bouts<br>· m1: 101 bouts<br>· m2: 166 bouts<br>· m3: 343 bouts<br>· m4: 226 bouts<br>· m5: 777 bouts<br>· m6: 1434 bouts<br>· m7: 998 bouts<br>· m8: 262 bouts<br>· m9: 28 bouts<br>· i1: 71 bouts<br>· i2: 189 bouts<br>· i3: 128 bouts<br>· i4: 101 bouts<br>· i5: 189 bouts<br>· i6: 80 bouts<br>· i7: 239 bouts<br>· i8: 229 bouts<br>· i9: 289 bouts	One-way ANOVA<br><br>Post-hoc test: Tukey-Kramer test	F(17,5832) = 20.81, **** P < 0.0001<br>m1 vs. m2: P > 0.9999<br>m1 vs. m3: ** P = 0.0054<br>m1 vs. m9: *** P = 0.0004<br>m1 vs. m4-m8, i1-i9: **** P < 0.0001<br>m2 vs. m3: *** P = 0.0010<br>m2 vs. m9: *** P = 0.0003<br>m2 vs. m4-m8, i1-i9: **** P < 0.0001<br>m3 vs. m4: P = 0.84<br>m3 vs. m5: P = 0.10<br>m3 vs. m6: P = 0.39<br>m3 vs. m7: P = 0.70<br>m3 vs. m8: P = 0.82<br>m3 vs. m9: P = 0.34<br>m3 vs. i1-i9: **** P < 0.0001<br>m4 vs. m5: P > 0.9999<br>m4 vs. m6: P > 0.9999<br>m4 vs. m7: P > 0.9999<br>m4 vs. m8: P > 0.9999<br>m4 vs. m9: P = 0.9351<br>m4 vs. i1-i9: **** P < 0.0001<br>m5 vs. m6: P = 0.9998<br>m5 vs. m7: P = 0.9943<br>m5 vs. m8: P > 0.9999<br>m5 vs. m9: P = 0.9615<br>m5 vs. i1-i9: **** P < 0.0001<br>m6 vs. m7: P > 0.9999<br>m6 vs. m8: P > 0.9999

			m6 vs. m9: P = 0.87<br>m6 vs. i1-i9: **** P < 0.0001<br>m7 vs. m8: P > 0.9999<br>m7 vs. m9: P = 0.82<br>m7 vs. i1-i9: **** P < 0.0001<br>m8 vs. m9: P = 0.92<br>m8 vs. i1-i9: **** P < 0.0001<br>i1 vs. i2: P = 0.97<br>i1 vs. i3: P = 0.41<br>i1 vs. i4: * P = 0.04<br>i1 vs. i5: P > 0.9999<br>i1 vs. i6: P > 0.9999<br>i1 vs. i7: P = 0.12<br>i1 vs. i8: P = 0.46<br>i1 vs. i9: ** P = 0.0027<br>i2 vs. i3: P > 0.9999<br>i2 vs. i4: *** P = 0.0008<br>i2 vs. i5: P = 0.9995<br>i2 vs. i6: P > 0.9999<br>i2 vs. i7: ** P = 0.0020<br>i2 vs. i8: ** P = 0.0082<br>i2 vs. i9: **** P < 0.0001<br>i3 vs. i4: **** P < 0.0001<br>i3 vs. i5: P = 0.85<br>i3 vs. i6: P = 0.9985<br>i3 vs. i7: **** P < 0.0001<br>i3 vs. i8: *** P = 0.0002<br>i3 vs. i9: **** P < 0.0001<br>i4 vs. i5: * P = 0.033<br>i4 vs. i6: * P = 0.018<br>i4 vs. i7: P > 0.9999<br>i4 vs. i8: P = 0.9792<br>i4 vs. i9: P > 0.9999<br>i5 vs. i6: P > 0.9999<br>i5 vs. i7: P = 0.098<br>i5 vs. i8: P = 0.38<br>i5 vs. i9: ** P = 0.0023<br>i6 vs. i7: P = 0.056<br>i6 vs. i8: P = 0.24<br>i6 vs. i9: ** P = 0.0013<br>i7 vs. i8: P > 0.9999<br>i7 vs. i9: P = 0.9997<br>i8 vs. i9: P = 0.74
Figure 3c	n = 32 mice<br>· Control: 15 mice<br>· Inhibition: 17 mice	Two-way repeated measures ANOVA<br>Factor 1: Time (repeated measure)<br>Factor 2: Optogenetic treatment (between subject)	(Demonstrator)<br>Factor 1: F(3.222, 96.65) = 105.9, **** P < 0.0001<br>Factor 2: F(1, 30) = 0.3874, P = 0.54<br>(Observer)<br>Factor 1: F(8.011, 240.3) = 15.96, **** P < 0.0001<br>Factor 2: F(1, 30) = 0.5345, P = 0.47
Figure 3i	n = 32 mice<br>· Control: 15 mice<br>· Inhibition: 17 mice	Unpaired t-test (two-sided)	Habituation period: t = 0.3702, P = 0.71<br>Conditioning period: t = 2.345, * P = 0.026

Figure 3j	n = 32 mice<br>· Control: 15 mice<br>· Inhibition: 17 mice	Unpaired t-test (two-sided)	Habituation period: t = -1.516, P = 0.14<br>Conditioning period: t = -2.069, * P = 0.047
Figure 3k	n = 32 mice<br>· Control: 15 mice<br>· Inhibition: 17 mice	Unpaired t-test (two-sided) using mean x coordinate during each period	Habituation period: t = 1.544, P = 0.13<br>Conditioning period: t = 2.498, * P = 0.018
Figure 4i	n = 4 mice	Paired t-test (two-sided)	t = 4.118, * P = 0.026
Figure 4n	n = 4 mice	Paired t-test (two-sided)	t = 15.30, *** P = 0.0006
Figure 5e	n = 355 cells / n = 4 mice<br>· Freezing positively correlated: 44 cells<br>· Freezing n.s.: 246 cells<br>· Freezing negatively correlated: 65 cells<br>· n = 60 bouts	Wilcoxon signed-rank test	-2-0 sec vs. 0-2 sec<br>· Pos., **** P < 0.0001<br>· n.s., **** P < 0.0001<br>· Neg., ** P = 0.0012
Figure 5f	n = 355 cells / n = 4 mice<br>· Freezing positively correlated: 44 cells<br>· Freezing n.s.: 246 cells<br>· Freezing negatively correlated: 65 cells	Kruskal-Wallis test<br><br>Post-hoc test: Dunn's multiple comparisons test	$\chi^2(2)$ = 27.28, **** P < 0.0001<br><br>Dunn's multiple comparisons test:<br>Pos. vs. n.s.: ** P = 0.001<br>Pos. vs. Neg.: ****P < 0.0001<br>n.s. vs. Neg.: *** P = 0.0009
Figure 5g	n = 355 cells / n = 4 mice<br>· Shock activated: 15 cells<br>· Shock n.s.: 312 cells<br>· Shock surppressed: 28 cells	Kruskal-Wallis test<br><br>Post-hoc test: Dunn's multiple comparisons test	$\chi^2(2)$ = 29.36, **** P < 0.0001<br><br>Dunn's multiple comparisons test:<br>Act. vs. n.s.: ** P = 0.0030<br>Act. vs. Sup.: ****P < 0.0001<br>n.s. vs. Sup.: *** P = 0.0001
Figure 6d	n = 384 cells / n = 4 mice<br>· Shock activated: 17 cells<br>· Shock n.s.: 337 cells<br>· Shock surppressed: 30 cells	Kruskal-Wallis test<br><br>Post-hoc test: Dunn's multiple comparisons test	$\chi^2(2)$ = 26.58, **** P < 0.0001<br><br>Dunn's multiple comparisons test:<br>Act. vs. n.s.: P = 0.91<br>Act. vs. Sup.: *** P = 0.0002<br>n.s. vs. Sup.: **** P < 0.0001
Figure 6f	n = 376 cells / n = 4 mice<br>· Freezing positively correlated: 43 cells<br>· Freezing n.s.: 273 cells<br>· Freezing negatively correlated: 60 cells	Kruskal-Wallis test<br><br>Post-hoc test: Dunn's multiple comparisons test	$\chi^2(2)$ = 16.14, *** P = 0.0003<br><br>Dunn's multiple comparisons test:<br>Pos. vs. n.s.: *** P = 0.0002<br>Pos. vs. Neg.: ** P < 0.0047<br>n.s. vs. Neg.: P > 0.99

Figure 6h	n = 384 cells / n = 4 mice<br>· Freezing positively correlated: 45 cells<br>· Freezing n.s.: 279 cells<br>· Freezing negatively correlated: 60 cells	Wilcoxon signed-rank test	-2-0 sec vs. 0-2 sec<br>· Pos., **** P < 0.0001<br>· n.s., P = 0.59<br>· Neg., P = 0.20
Figure 6l	n = 360 cells / n = 4 mice<br>· Shock activated: 18 cells<br>· Shock n.s.: 327 cells<br>· Shock surppressed: 15 cells	Kruskal-Wallis test<br><br>Post-hoc test: Dunn's multiple comparisons test	$\chi^2(2) = 7.689$, * P < 0.021<br><br>Dunn's multiple comparisons test:<br>Act. vs. n.s.: * P = 0.023<br>Act. vs. Sup.: P = 0.061<br>n.s. vs. Sup.: P > 0.99
Figure 6n	n = 356 cells / n = 4 mice<br>· Freezing positively correlated: 40 cells<br>· Freezing n.s.: 211 cells<br>· Freezing negatively correlated: 105 cells	Kruskal-Wallis test<br><br>Post-hoc test: Dunn's multiple comparisons test	$\chi^2(2) = 13.51$, *** P = 0.0012<br><br>Dunn's multiple comparisons test:<br>Pos. vs. n.s.: P = 0.13<br>Pos. vs. Neg.: ** P = 0.0014<br>n.s. vs. Neg.: * P = 0.034
Figure 6p	n = 360 cells / n = 4 mice<br>· Freezing positively correlated: 40 cells<br>· Freezing n.s.: 215 cells<br>· Freezing negatively correlated: 105 cells	Wilcoxon signed-rank test	-2-0 sec vs. 0-2 sec<br>· Pos., **** P < 0.0001<br>· n.s., * P = 0.021<br>· Neg., P = 0.15
Figure 7c	n = 35 mice<br>· Control: 19 mice<br>· Inhibition: 16 mice	Two-way repeated measures ANOVA<br>Factor 1: Time (repeated measure)<br>Factor 2: Optogenetic treatment (between subject)	(Demonstrator)<br>Factor 1: F(3.032,100) = 126.1, P < 0.0001<br>Factor 2: F(1, 33) = 1.425, P = 0.24<br>(Observer)<br>Factor 1: F(14,462) = 12.33, P < 0.0001<br>Factor 2: F(1, 33) = 1.049, P = 0.31
Figure 7f	n = 24 mice<br>· Control: 12 mice<br>· Inhibition: 12 mice	Two-way repeated measures ANOVA<br>Factor 1: Time (repeated measure)<br>Factor 2: Optogenetic treatment (between subject)	(Demonstrator)<br>Factor 1: F(6.185, 136.1) = 393.2, P < 0.0001<br>Factor 2: F(1, 22) = 2.055, P = 0.17<br>(Observer)<br>Factor 1: F(7.326, 161.2) = 16.09, P < 0.0001<br>Factor 2: F(1, 22) = 0.1431, P = 0.71
Figure 7j	n = 35 mice<br>· Control: 19 mice<br>· Inhibition: 16 mice	Unpaired t-test (two-sided)	Control: t = 0.4903, P = 0.63<br>Inhibition: t = 2.213, * P = 0.043
Figure 7k	n = 24 mice<br>· Control: 12 mice<br>· Inhibition: 12 mice	Unpaired t-test (two-sided)	Control: t = 0.5920, P = 0.57<br>Inhibition: t = 2.553, * P = 0.027
