# Supplementary material for: Ventromedial prefrontal neurons represent self-states shaped by vicarious fear in male mice
Source: Nat Commun. 2023 Jul 3;14:3458. doi: 10.1038/s41467-023-39081-5 (PMC10318047; doi:10.1038/s41467-023-39081-5)
Supplement: Supplementary file 3 — Reporting Summary [file 41467_2023_39081_MOESM3_ESM.pdf]

nature portfolio

Corresponding author(s): Teruhiro Okuyama

Last updated by author(s): Apr 20, 2023

Reporting Summary

Nature Portfolio wishes to improve the reproducibility of the work that we publish. This form provides structure for consistency and transparency in reporting. For further information on Nature Portfolio policies, see our Editorial Policies and the Editorial Policy Checklist.

Statistics

For all statistical analyses, confirm that the following items are present in the figure legend, table legend, main text, or Methods section.

n/a	Confirmed	
☐	☒	The exact sample size (*n*) for each experimental group/condition, given as a discrete number and unit of measurement
☐	☒	A statement on whether measurements were taken from distinct samples or whether the same sample was measured repeatedly
☐	☒	The statistical test(s) used AND whether they are one- or two-sided<br>*Only common tests should be described solely by name; describe more complex techniques in the Methods section.*
☐	☒	A description of all covariates tested
☐	☒	A description of any assumptions or corrections, such as tests of normality and adjustment for multiple comparisons
☐	☒	A full description of the statistical parameters including central tendency (e.g. means) or other basic estimates (e.g. regression coefficient) AND variation (e.g. standard deviation) or associated estimates of uncertainty (e.g. confidence intervals)
☐	☒	For null hypothesis testing, the test statistic (e.g. F, t, r) with confidence intervals, effect sizes, degrees of freedom and P value noted<br>*Give P values as exact values whenever suitable.*
☒	☐	For Bayesian analysis, information on the choice of priors and Markov chain Monte Carlo settings
☒	☐	For hierarchical and complex designs, identification of the appropriate level for tests and full reporting of outcomes
☐	☒	Estimates of effect sizes (e.g. Cohen's *d*, Pearson's *r*), indicating how they were calculated

Our web collection on statistics for biologists contains articles on many of the points above.

Software and code

Policy information about availability of computer code

Data collection	FreezeFrame version 4 and 5 (Actimetrics)<br>64-channel silicon probe (A4×16-Poly2–5 mm-20s-150-160, NeuroNexus)<br>Open Ephys Data Acquisition System (Open Ephys)<br>Twist-on eFocus fluorescence microscope (Doric)<br>Doric Neuroscience Studio 5.4.1.5 (Doric)<br>FV3000 (Olympus)<br>BZ-X710 (Keyence)
Data analysis	MATLAB R2020a (Windows), R2021a (MacOS), and R2022a (MacOS) (Matworks)<br>ImageJ2 2.9.0 (NIH)<br>Prism 9.1.2 (GraphPad)<br>DeepLabCut 2.1.10 (http://www.mackenziemathislab.org/deeplabcut)<br>Doric Neuroscience Studio 5.4.1.5 (Doric)<br>EXTRACT 0.7.3 (https://github.com/schnitzer-lab/EXTRACT-public)<br>FreezeFrame version 4 and 5 (Actimetrics)<br>Watershed clustering code (https://github.com/murthylab/pulseTypePipeline)<br>raacampbell/shadedErrorBar (https://jp.mathworks.com/matlabcentral/fileexchange/26311-raacampbell-shadederrorbar)<br>Kilosort2 (https://github.com/jamesjun/Kilosort2)<br>Huang et al., 2023 (https://github.com/okuyamalab/Huang_et_al_2023_NatCommun)

For manuscripts utilizing custom algorithms or software that are central to the research but not yet described in published literature, software must be made available to editors and reviewers. We strongly encourage code deposition in a community repository (e.g. GitHub). See the Nature Portfolio guidelines for submitting code & software for further information.

Data

Policy information about availability of data

All manuscripts must include a data availability statement. This statement should provide the following information, where applicable:

- Accession codes, unique identifiers, or web links for publicly available datasets
- A description of any restrictions on data availability
- For clinical datasets or third party data, please ensure that the statement adheres to our policy

The source data underlying all Figures are provided as Source Data files. All data reported in this study will be shared by the lead contact upon request.

Human research participants

Policy information about studies involving human research participants and Sex and Gender in Research.

Reporting on sex and gender	Not applicable to this study
Population characteristics	Not applicable to this study
Recruitment	Not applicable to this study
Ethics oversight	Not applicable to this study

Note that full information on the approval of the study protocol must also be provided in the manuscript.

Field-specific reporting

Please select the one below that is the best fit for your research. If you are not sure, read the appropriate sections before making your selection.

☒ Life sciences ☐ Behavioural & social sciences ☐ Ecological, evolutionary & environmental sciences

For a reference copy of the document with all sections, see nature.com/documents/nr-reporting-summary-flat.pdf

Life sciences study design

All studies must disclose on these points even when the disclosure is negative.

Sample size	No statistical methods were used to determine sample sizes. Suitable sample sizes were determined based on our previous experiences and similar studies which are generally employed in the field of study: Okuyama et al., Science (2016); Jeon et al., Nat Neurosci (2010); Pisansky et al., Nat Commun (2017).
Data exclusions	All mice used for optogenetic inhibition and microendoscopy experiments were perfused and blindly post-hoc verified to include only individuals with appropriate expression and accurate optic fiber or GRIN lens implant position for further analysis. Only individuals who demonstrated less than 50% freezing during the habituation period (0–300 s) were included in the microendoscopy analysis. During microendoscopy, two mice had missing microendoscopy image frames (ACC-vmPFC mouse #10 [3 frames:0.12 s], BLA-vmPFC mouse #14 [30 frames:1.2 s]) at the end of the task due to technical problems. The last bouts from these individuals were omitted from the statistical analysis. To calculate shock correlation, a cell must show at least one calcium event during the conditioning period (301–900 s). Owing to the absence of calcium events during the conditioning period, four cells from the vmPFC group (Fig. 5g) and four cells from the BLA-vmPFC group (Fig. 6l) were excluded from the data analysis.
Replication	We showed representative images and described the exact n values in the corresponding legends. All behavioral experiments were conducted in at least three different batches, and all batches showed similar trends.
Randomization	All the subject mice were randomly assigned to each experimental groups in each study. The nonlittermate demonstrator mice were randomly assigned to the observer mice.
Blinding	All the behavioral experiments were conducted with a blind group allocation during data collection and analysis, except for the in vivo Ca2+ imaging experiments due to its high complexity. All animal behaviors were automatically tracked using DeepLabCut.

Reporting for specific materials, systems and methods

We require information from authors about some types of materials, experimental systems and methods used in many studies. Here, indicate whether each material, system or method listed is relevant to your study. If you are not sure if a list item applies to your research, read the appropriate section before selecting a response.

Materials & experimental systems

n/a	Involved in the study
☐	☒ Antibodies
☒	☐ Eukaryotic cell lines
☒	☐ Palaeontology and archaeology
☐	☒ Animals and other organisms
☒	☐ Clinical data
☒	☐ Dual use research of concern

Methods

n/a	Involved in the study
☒	☐ ChIP-seq
☒	☐ Flow cytometry
☒	☐ MRI-based neuroimaging

Antibodies

Antibodies used	Primary antibodies: chicken anti-GFP antibody (1:1000, A10262, Thermo Fisher Scientific), rabbit anti-RFP antibody (1:1000, 600-401-379, Rockland)<br>Secondary antibodies: anti-chicken Alexa Fluor-488 conjugated secondary antibody (1:500, A11039, Thermo Fisher Scientific), anti-rabbit Alexa Fluor-546 conjugated secondary antibody (1:500, A11010, Thermo Fisher Scientific)
Validation	The antibodies used in this study are validated by the manufacturers as shown in the following websites.<br>chicken anti-GFP antibody: https://www.thermofisher.com/antibody/product/GFP-Antibody-Polyclonal/A10262<br>rabbit anti-RFP antibody: https://www.rockland.com/categories/primary-antibodies/rfp-antibody-pre-adsorbed-600-401-379/<br>anti-chicken Alexa Fluor-488 conjugated secondary antibody: https://www.thermofisher.com/antibody/product/Goat-anti-Chicken-IgY-H-L-Secondary-Antibody-Polyclonal/A-11039<br>anti-rabbit Alexa Fluor-546 conjugated secondary antibody: https://www.thermofisher.com/antibody/product/Goat-anti-Rabbit-IgG-H-L-Cross-Adsorbed-Secondary-Antibody-Polyclonal/A-11010

Animals and other research organisms

Policy information about studies involving animals; ARRIVE guidelines recommended for reporting animal research, and Sex and Gender in Research

Laboratory animals	The study was conducted using male C57BL/6J (B6) mice obtained from Clea Japan. All animals were housed in the Institute for Quantitative Biosciences facility under a 12 h (7 am – 7 pm) light/dark cycle, 23 ± 2 °C, 50 % humidity with food and water ad libitum. Twelve- to 20-week-old male mice were used as observers and demonstrators.
Wild animals	No wild animals were used in this study.
Reporting on sex	Only male mice were used in this study.
Field-collected samples	No field-collected samples were used in this study.
Ethics oversight	All procedures were performed in accordance with protocols approved by the Institutional Animal Care and Use Committee at the Institute for Quantitative Biosciences, the University of Tokyo (Protocol number 2915 (2018), 3112 (2019), 0201 (2020), 0314 (2021), 0403-2 (2022), A2022IQB018 (2023)).

Note that full information on the approval of the study protocol must also be provided in the manuscript.
